# Experimental Study on the Effect of Allogeneic Endothelial Progenitor Cells on Wound Healing in Diabetic Mice

**DOI:** 10.1155/2021/9962877

**Published:** 2021-10-21

**Authors:** Min Leng, Ying Peng, Manchang Pan, Hong Wang

**Affiliations:** ^1^Department of Burns, The Second Affiliated Hospital, Kunming Medical University, 374 Dian Burma Road, Wuhua District, Kunming 650000, China; ^2^Department of Burns and Plastic, Dazhou Central Hospital, 56 Nanyuemiao Street, Tongchuan District, Dazhou 635000, China; ^3^The First Affiliated Hospital, Kunming Medical Uiversity, 1168 Chunrong West Road, Yuhua Street, Kunming 650000, China; ^4^Department of Burns, The Changzhou Geriatric Hospital Affiliated with Soochow University, Changzhou 213000, China

## Abstract

Endothelial progenitor cells (EPCs) are involved in the neovascularization in traumatic and ischemic sites, but EPCs are “detained” in bone marrow under diabetic conditions, which results in reduction of the number of EPCs and their biological activity in peripheral blood. Based on our previous study to mobilize autologous bone marrow EPCs by administering AMD3100+G-CSF to realize the optimal effect, our present study is aimed at exploring the effects of transplanting EPCs locally in a wound model of diabetic mice. First, we prepared and identified EPCs, and the biological functions and molecular characteristics were compared between EPCs from DB/+ and DB/DB mice. Then, we performed full-thickness skin resection in DB/DB mice and tested the effect of local transplantation of EPCs on skin wound healing. The wound healing process was recorded using digital photographs. The animals were sacrificed on postoperative days 7, 14, and 17 for histological and molecular analysis. Our results showed that DB/+ EPCs were biologically more active than those of DB/DB EPCs. When compared with the control group, local transplantation of EPCs accelerated wound healing in DB/DB mice by promoting wound granulation tissue formation, angiogenesis, and collagen fiber deposition, but there was no significant difference in wound healing between DB/+ EPCs and DB/DB EPCs transplanted into the wound. Furthermore, local transplantation of EPCs promoted the expression of SDF-1, CXCR4, and VEGF. We speculated that EPC transplantation may promote wound healing through the SDF-1/CXCR4 axis. This point is worth exploring further. Present data are of considerable significance because they raise the possibility of promoting wound healing by isolating autologous EPCs from the patient, which provides a new approach for the clinical treatment of diabetic wounds in the future.

## 1. Introduction

Diabetes is a common endocrine disease, and approximately 20% of patients develop diabetic wounds, with leg or foot ulcers being the most common [1]. The decrease in the ability to metabolize glucose in patients can cause hyperglycemia, which seriously affects the process of wound repair [2]. Moreover, due to the lack of effective prevention and control measures at present, the incidence of delayed healing of diabetic patients is on the rise worldwide, which brings a huge economic burden to patients and the society. Based on WHO epidemiological projections, diabetes will become the seventh leading cause of death by 2030. More than 80% of diabetes deaths will occur in low- and middle-income countries [1–3], while approximately 50%-70% of amputations are due to diabetes trauma. It has been reported that leg amputation occurs every 30 seconds due to diabetes trauma worldwide [4].

Wound healing is a complex biological process involving a variety of cells and cytokines [5]. Chronic surface wounds (commonly known as ulcers), which are difficult to heal, are also called chronic wounds. Epidemiological studies have shown that more than 1/3 of chronic refractory wounds are caused by diabetes [6]. Diabetes can lead to impaired wound healing by affecting one or more biological mechanisms in the process of wound healing. These lesions are usually caused by hyperglycemia, chronic inflammation, dysfunction of the microcirculatory system or macrocirculatory system, hypoxia, neuropathy, or impaired neuropeptide signaling [1, 7]. Studies have shown that delayed healing of diabetic wounds is mainly related to changes in cellular and biological factors and their activities. Typically, almost all cells involved in diabetic wound healing were severely damaged and did not respond well to conventional growth factors or drug therapy. To overcome these shortcomings, cell therapy has been extensively studied. In addition to directly participating in the regeneration as diabetic wound repairing cells, they can also provide paracrine or nutritional signals to change the wound microenvironment into a state that promotes regeneration. Cell therapy may help to improve immune regulation, cell differentiation, angiogenesis, production of extracellular matrix (ECM), and growth factors and ultimately promote wound repair [8].

Endothelial progenitor cells (EPCs), which normally exist in the bone marrow and are rarely found in the peripheral blood, are heterogeneous cells that can differentiate into endothelial cells (ECs) [9]. EPCs migrate out of the BM into the peripheral blood under the stimulation of various factors. This process, called EPC mobilization, is a complex process and not well understood [10]. It can be achieved through divergent functions of a single factor or synergistic effects of multiple factors. Although the mechanism of EPC mobilization remains unclear, there is strong evidence that EPCs can promote endothelial repair by mobilization out of the BM or exogenous transplantation to the ischemic tissue. Transplantation of EPCs in ischemic animal models can promote neovascularization [11]. Intravascular perfusion of EPCs can improve the clinical outcome in patients with acute myocardial infarction (AMI) [12]. In recent years, transplantation of exogenous EPCs has also been found to have a positive effect on wound healing [13, 14]. EPCs from the BM mediate the reendothelialization process after vascular injury through multiple steps, such as mobilization, chemotaxis, homing, proliferation, and differentiation [15, 16]. The activation of EPCs is the first step in this process and is strictly regulated. After activation, EPCs secrete a variety of factors to promote the cell proliferation, migration, and tube formation of mature ECs and other peripheral blood EPCs; thereby, they promote overall wound healing [17, 18].

Since angiogenesis failure is an important cause of the difficulty of healing diabetic wounds, promoting the construction of new blood vessels has become a research direction in the treatment of diabetic wounds, and vascular EPCs are the key factor in angiogenesis. Recent studies have shown that the number and function of vascular EPCs in diabetic patients are impaired [19]. Studies have confirmed that EPC transplantation can promote angiogenesis in the ischemic hind limbs in diabetic mice [20]. Jeong et al. [21] found that the sensory and motor conduction velocity, blood flow, and capillary density of the sciatic nerve in rats were restored by local injection of EPCs into the hind limbs of diabetic neuropathy rats. Additionally, studies have shown that a variety of factors that regulate cell functions, such as VEGF or SDF-1, can play crucial roles in vascular repair [22, 23]. Under normal circumstances, the production and secretion of VEGF is increased in cells such as ECs or platelets. SDF-1 is viewed as the most important chemokine for EPCs, and a high local SDF-1 content is conducive to the chemotaxis and migration of cells to the injured site [24]. Fan et al. found that SDF-1*α* could increase the number of EPCs by upregulating the expression of microRNA-126 and microRNA-125b in animal experiments [25]. In addition, upregulation of CXCR4 expression in ischemic tissues has also been proven to promote EPC homing, neovascularization, and adhesion of EPC to injured vessels and consequently promote angiogenesis at the wound site [26, 27]. Therefore, SDF-1, VEGF, and CXCR4 are viewed as three important factors for EPCs to exert biological effects at the wound site. In conclusion, in addition to increasing the number of EPCs on the wound surface through transplantation, the chemotaxis and migration of cells can also effectively accelerate wound healing. Therefore, EPC transplantation is expected to be an effective method for the treatment of diabetic refractory wounds [28]. In this study, we aimed to explore whether locally EPC transplantation could effectively promote wound healing and its potential signal pathway.

## 2. Materials and Methods

### 2.1. Mobilization of EPCs

DB/+ (body weight 20 ± 2 g) and DB/DB (body weight 30 ± 2 g) male mice aged 5 to 6 weeks were purchased from Changzhou Cavens Laboratory Animal Co., Ltd. (Changzhou, China). Following the optimal EPC mobilization method reported before [28], each DB/DB mouse first received a single subcutaneous injection of AMD3100 6 mg/(kg d) 1 d in the left hind limb, followed by continuous subcutaneous injection of G-CSF (Qilu Pharmaceutical Co., Ltd.). Each DB/+ mouse was injected subcutaneously with G-CSF in the left hind limb for 5 days followed by a single injection of AMD3100 for 1 d.

### 2.2. EPC Separation and Expansion

The peripheral blood was collected by cardiac puncture on day 10 after mobilization from DB/DB mice and on day 7 from DB/+ mice, according to the effective EPC mobilization method explored previously [29]. After anesthesia with 1% pentobarbital sodium, a needle attached to a 2 ml syringe containing heparin was inserted into the chest of a mouse where the apical pulsation of the heart was externally visible, and blood was collected in a tube with heparin as anticoagulant. Mononuclear cells were separated by density gradient centrifugation, washed twice with PBS, and suspended in EGM-2 (Lonza) containing 10% fetal bovine serum (Gibco) and 1% penicillin-streptomycin solution. Finally, the cells were seeded in a 100 mm petri dish and placed at 37°C in the presence of 5% CO_2_ for culturing. When the cells reached 80% confluence, trypsin (Gibco) was added for passaging, and the second-generation DB/+ and DB/DB EPCs were collected for the following assays.

### 2.3. Flow Cytometric Analysis

After digestion, DB/+ and DB/DB EPCs were resuspended in 100 *μ*l staining buffer and stained with appropriate amounts of fluorescently labeled antibodies against surface markers (PE-CY7 anti-mouse CD34, APC anti-mouse CD133, and PE anti-mouse CD309). Cells were stained at room temperature in the dark for 30 min. Cells were then washed and analyzed using a Guava easyCyte^™^ flow cytometer (Millipore, Billerica, USA).

### 2.4. Dil-Ac-LDL Uptake and FITC-UEA-1 Staining

Second-generation DB/+-EPCs and DB/DB-EPCs were incubated at room temperature with 10 *μ*g/ml Dil-Ac-LDL (Biomedical Technologies, Stoughton, MA) for 4 h and then fixed in 4% paraformaldehyde for 15 min. The fixed cells were washed twice in PBS (pH 7.4). Subsequently, cells were incubated for 1 h with 10 *μ*g/ml FITC-UEA-1 (Molecular Probes, USA). The images of fluorescently labeled cells were captured using a laser scanning confocal microscope (Leica, Germany). Cells positive for both markers are considered EPCs [30].

### 2.5. Cell Migration

Transwell inserts were placed into 24-well plates (Corning Transwell, Lowell, MA). Fifty thousand (5 × 10^4^) EPCs from DB/+ and DB/DB mice were plated in the upper chambers. Medium containing VEGF (50 ng/ml) was placed in the lower chambers. Cells were incubated at 37°C for 24 h. After incubation, cells were fixed in 2% paraformaldehyde and stained with crystal violet dye (Sigma-Aldrich, St. Louis, MO). Four random fields were scanned and analyzed by ImageJ. A bar graph of the sample pixel intensities was generated to show the results, which were reported as the means and standard deviations.

### 2.6. Angiogenesis Assay

A hundred thousand (1 × 10^5^) DB/+ and DB/DB EPCs were inoculated into 6-well plates precoated with Matrigel (BD Biosciences, Bedford, MA, USA). After incubation for 8 h, an inverted phase contrast microscope (Laika Microsystems, Germany) was used for observation. Six microscopic fields (100-fold magnification) were randomly selected to acquire images of the tubes and determine the neovascularization of the EPCs.

### 2.7. Preparation and Treatment of Diabetic Wound Model

**Figure 1:**
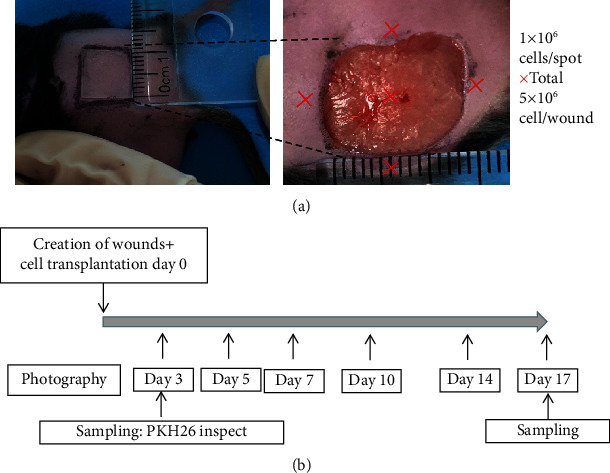
Generation of the mouse wound model and the wound observation schedule. (a) A full-thickness skin defect (size 1 × 1 cm^2^) was formed on the back of each mouse. EPCs were subcutaneously transplanted at four sites in each wound, with 0.5 × 10^6^ cells at each site. (b) After the formation of the wound and the transplantation of EPCs, the wound was photographed at specified time points. On days 3, 7, 14, and 17, the wound was excised, and tissue was collected.

**Figure 2:**
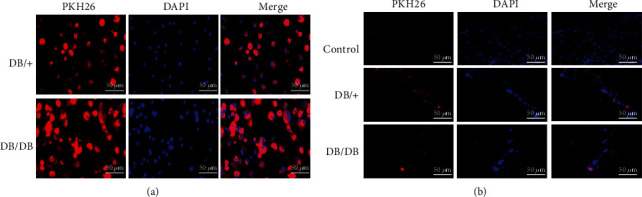
Fluorescent tracing of DB/DB EPCs and DB/+ EPCs. (a) DB/DB EPCs and DB/+ EPCs were labeled with PKH26 before transplantation. (b) After 3 days of treatment, frozen slices were prepared from wounds of three groups of mice and observed under a fluorescence microscope. Red fluorescence of EPCs was visible in the DB/DB EPC-treated group and DB/+ EPC-treated group. No red fluorescence was observed in the control group.

A total of 36 male DB/DB mice (8–10 weeks old; weight 40–45 g) were purchased from Changzhou Kavins Laboratory Animal Co., Ltd. After anesthesia with 1% pentobarbital sodium, back was depilated and disinfected. Then, a skin incision was created with a scalpel/bistoury, and subcutaneous tissue was dissected with surgical scissors to make a full-thickness skin defect wound with an area of 1 cm × 1 cm under aseptic conditions (Figure 1(a)). The mice were randomly divided into three groups of 12 mice each. Mice in the two EPC treatment groups were injected with second-generation PKH26-labeled EPCs (a total of 2 × 10^6^/200 *μ*l [31], Figure 2) on the four corners of the wound, while the control group was injected with the same amount of normal saline.

### 2.8. Wound Contraction Assessment

After transplantation, wounds were photographed on days 0, 3, 5, 7, 10, 14, and 17 (Figure 1(b)). From a sufficient distance to cover the entire area of the injury, with the lens pointing parallel to the wound, an 8-megapixel digital camera (Canon USA, Melville, NY, USA) was used to take the wound image. Image-Pro Plus 6.0 (GraphPad Software Inc., La Jolla, CA, USA) was used to measure the wound area, and the percentage of wound closure was calculated as the wound closure rate (%) = [(wound area on day 0 − wound area on day *N*)/wound area on day 0] × 100. Wound tissues were harvested from anesthetized mice on postoperative days 3, 7, 14, and 17 [32]. The wound tissue was removed from the surface layer, including the muscle layer. Excision was performed outside the margin of the original wound. Each sample was vertically divided into four blocks.

### 2.9. H&E and Masson Staining

The harvested wound tissue was fixed in 4% paraformaldehyde (PFA; 3M). The fixed tissue was embedded in paraffin according to a standard protocol, and the tissue paraffin block was cut to a thickness of 4 *μ*m. For histological analysis, the sections were stained with hematoxylin and eosin (Sigma-Aldrich; Merck KGaA). Image-Pro Plus 6.0 (GraphPad Software Inc., La Jolla, CA, USA) was used to acquire the images. According to the published data on wound healing in similar experimental models, this study used the histologic score for evaluation [33]. The margins of the wound in each of the sections and normal control wounds were used as the baseline for scoring (Table 1). For Masson staining, the stained sections were observed using a 200-fold normal microscope. The pixelated region was measured using Image-Pro Plus 6.0, and the mature collagen percentage was calculated by dividing the blue region (mature collagen) by the light blue to strong blue region (total collagen region).

### 2.10. CD31 Immunohistochemical Staining Analysis

Paraffin sections (4 *μ*m) were deparaffinized and rehydrated. The rehydrated sections were subsequently subjected to antigen retrieval by boiling. Rabbit-derived CD31 antibody (1 : 300) and enzyme-labeled sheep anti-mouse/rabbit IgG polymer were added for immunohistochemical staining. Microangiogenesis was observed under a 200-fold-positive microscope, and the positive staining was brown. Three views from each image were obtained, and Image-Pro Plot 6.0 (American Media Cybernetics Company) software was used for analysis. CD31 = positive area + total visual field × 100%.

### 2.11. qRT-PCR

EPCs from DB/+ and DB/DB mice and the 7-, 14-, and 17-day wound tissues were used for qRT-PCR. Total RNA was extracted using TRIzol reagent (RNA STAT 60, Tel-Test Inc., Austin, TX). Primers for VEGF (MQP024363), SDF-1 (MQP024071), and CXCR4 (MQP091536) were purchased from Thermo Fisher Scientific (Waltham, MA). qRT-PCR was performed in duplicate in a 20 *μ*l reaction using the ABI 7500 qRT-PCR system (Bio-Rad, USA). The comparative cycle time (Ct) method was used to determine fold differences between samples, and the amount of target genes was normalized to *β*-actin as an endogenous reference (2^−ΔΔCt^). The primers used for qRT-PCR are listed in Table 2.

### 2.12. Western Blot Assay

EPCs from DB/+ and DB/DB mice and the 7-, 14-, and 17-day wound tissues were used for analysis. Proteins were isolated with lysis buffer (Roche Diagnostic) containing protease inhibitors. The proteins were separated by SDS-PAGE and transferred onto nitrocellulose membranes. The membranes were blocked by incubating with 5% dry milk for 30 min and then incubated with antibodies against VEGF (1 : 1000; Millipore/ID: ABS82), SDF-1 (1 : 1000; Millipore/ID: GTX116092), and CXCR4 (1 : 1000; Millipore/ID:). *β*-Actin (1 : 5000, Abcam/ID: GTX22074, UAS) was used to normalize protein loading. After thorough washes, membranes were incubated with IgG (1 : 5000, Sigma) for one hour at RT.

### 2.13. Statistical Analysis

Independent Student's *t*-test was used to assess differences between different groups. The data are reported as the means ± standard deviation, where *P* < 0.05 demonstrated a significant difference between the two groups.

## 3. Results

### 3.1. Characterization of EPCs

**Figure 3:**
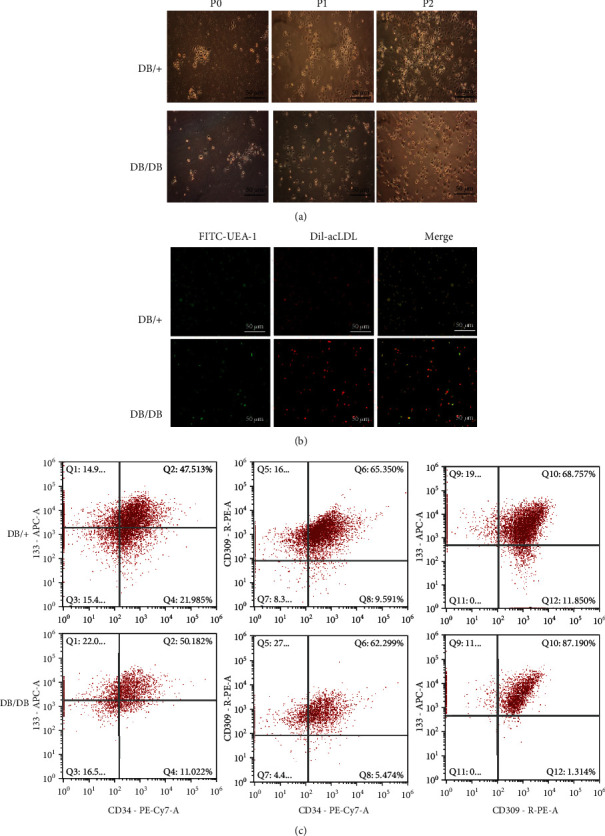
Phenotypic characterization of DB/DB EPCs and DB/+ EPCs. (a) DB/DB EPCs and DB/+ EPCs were cultured, and cells grew uniformly with cobblestone-like appearance. (b) Double-staining identification of EPCs. Dil-Ac-LDL-labeled cells showed red fluorescence, FITC-UEA-1-labeled cells showed green fluorescence, and the double-stained EPCs appeared yellow. The percentages of double-positive FITC-UEA-1/Dil-Ac-LDL cells were DB/+(92.23%) and DB/DB (89.72%). (c) Analyses of DB/DB EPC and DB/+ EPC surface markers CD34, CD133, and CD309 by flow cytometry. Three independent experiments were performed.

Since our study is aimed at exploring the effect of EPC transplantation on wound healing, we first attempted to isolate and identify EPCs. First, DB/+ and DB/DB EPCs were cultured in dishes coated with 0.1% gelatin (BI). We observed that both types of primary cells formed heterogeneous clones, which showed cobblestone appearance and proliferated significantly (Figure 3(a), left column). At the initial stage of P0 generation isolation, the cells were round. After 7 days of culture, colony growth appeared and the number increased gradually. After 14 days of culture, the cells showed short spindle and triangular morphology. Cells cultured for 21 days showed typical “paving stone”-like changes. Generations P1 and P2 showed short shuttle, triangular, and “paving stone” cell morphology. Regardless of generation P1 and generation P2, both EPCs with cobblestone-like morphology proliferated uniformly (Figure 3(a), middle and right columns). The second-generation DB/+ and DB/DB EPCs were both positive for Dil-Ac-LDL and FITC-UEA-1 staining (Figure 3(b)). Flow cytometry showed that both DB/+ and DB/DB EPCs were positive for CD34, CD309, and CD133 (Figure 3(c)). These characteristics are consistent with the EPCs described earlier [34].

### 3.2. Functions of EPCs

**Figure 4:**
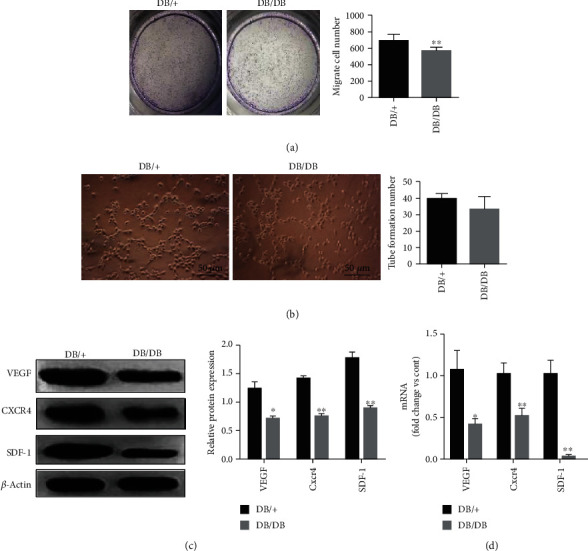
Biological function tests of DB/DB EPCs and DB/+ EPCs. (a) Transwell assays of cell migration of DB/DB EPCs and DB/+ EPCs (×2). The number of EPCs migrated within each field of vision (×2). (b) Tube formation assays of DB/DB EPCs and DB/+ EPCs. Images of tubes formed by EPCs cultured for 8 h (×20) and tube length quantification (×20). (c, d) VEGF, SDF-1, and CXCR4 mRNA expression (right) and protein expression (left, middle) by DB/DB EPCs and DB/+ EPCs. DB/DB EPCs were compared with DB/+ EPCs, ^∗^*P* < 0.05 and ^∗∗^*P* < 0.01.

To explore the functions of EPCs in wound healing, we examined biological functions of EPCs and three important factors related to their function: SDF-1, VEGF, and CXCR4. In the Transwell assay, when cell migration was observed after crystal violet staining under the microscope, the number of DB/+ EPCs was significantly higher than that of DB/DB EPCs, and the difference was statistically significant (*P* < 0.05) (Figure 4(a)). In the tube forming experiment, the tube length of the two types of EPCs was observed under a microscope. Our results showed that the tube length of DB/+ EPCs was longer than that of DB/DB EPCs (*P* < 0.05) (Figure 4(b)). In addition, qRT-PCR and western blot experiments were performed on the two types of EPCs. The mRNA expression levels of VEGF, SDF-1, and CXCR4 in DB/+ EPCs were significantly higher than those in DB/DB EPCs (*P* < 0.05 or 0.01). The protein expression levels of VEGF, SDF-1, and CXCR4 in DB/+ EPCs were also significantly higher than those in DB/DB EPCs (*P* < 0.05 or 0.01) (Figures 4(c) and 4(d)). These results showed that DB/+ had more robust biological functions than DB/DB EPCs.

### 3.3. Postoperative Wound Observation and Healing Rate

**Figure 5:**
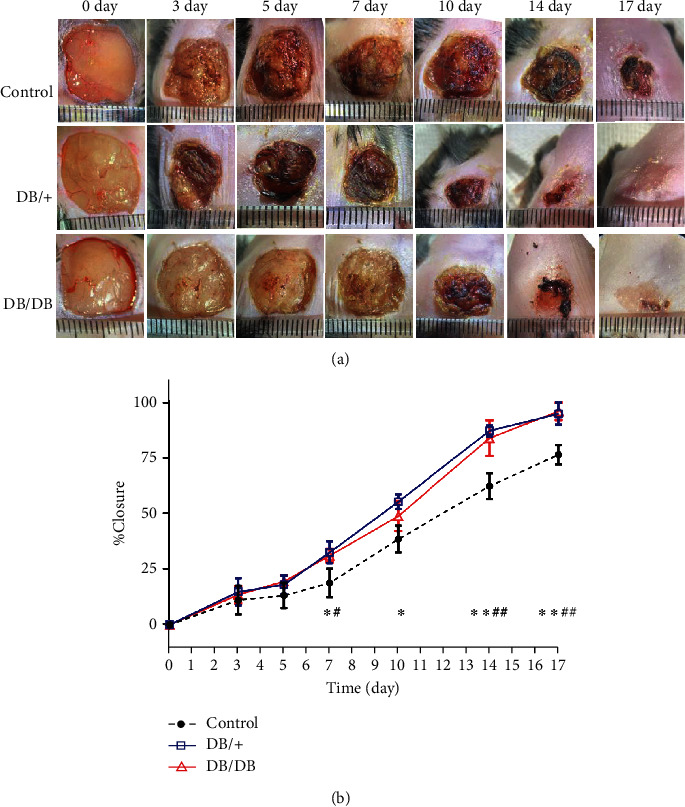
Comparison of wound closure rates between the EPC-treated groups and the control group. (a) Images of the wound at the specified time. Wounds in the EPC-treated groups tended to epithelialize faster than those in the control group. (b) The wound areas were photographed on days 0, 3, 5, 7, 10, 14, and 17. The wound area was measured by photogrammetry. Wound closure rate (%) = [(wound area on day 0 − wound area on day *N*)/wound area on day 0] × 100. When the DB/+ EPC-treated group was compared with the control group, ^∗^*P* < 0.05 and ^∗∗^*P* < 0.01; when the DB/DB EPC-treated group was compared with the control group, ^#^*P* < 0.05 and ^##^*P* < 0.01.

To investigate the effect of EPC transplantation on the wound healing rate, the wound was photographed at various times after local transplantation, and the wound area was measured. Compared with the control group, the wounds treated with the two types of EPCs both achieved significant wound area reduction with better closure (Figure 5(a)). On the 3rd and 5th days after injury, the wound healing rate of mice in the 3 groups was comparable (*P* > 0.05). Ten days after injury, the wound healing rate of the DB/DB EPC-treated group was similar to that of the control group (*P* > 0.05). The wound healing rate in both the DB/+ EPC- and DB/DB EPC-treated groups was significantly better than that in the control group at other time points (*P* < 0.05 or 0.01). At each time point after injury, the wound healing rate of mice in the DB/+ EPC-treated group was comparable to that in the DB/DB EPC-treated group (*P* > 0.05) (Figure 5(b)).

### 3.4. Promotion of Epithelialization and Deposition of Collagen on the Wound Surface by EPC Transplantation

**Figure 6:**
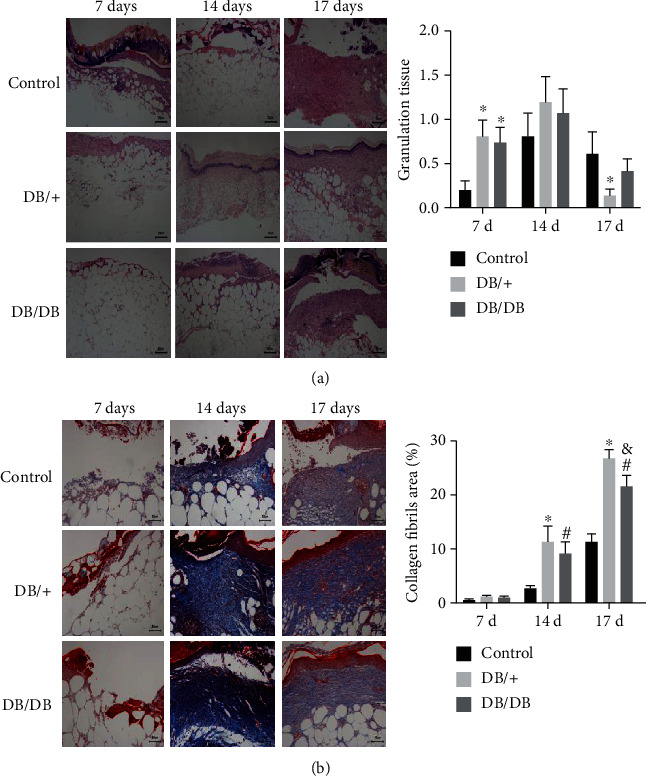
Histological analyses comparing EPC-treated groups and the control group. (a) Histological photomicrographs of the wounds (H&E staining) in the DB/DB EPC-treated, DB/+ EPC-treated, and the control groups (×40). Extensive reepithelialization, thicker granulation tissue, and many functional erythrocyte-containing vessels were observed in the EPC-treated groups. The histological scores of DB/+ and DB/DB EPC-treated wounds were significantly higher than those of control wounds on day 7, while there was no difference between the two EPC-treated groups. (b) Visualization and quantification of mature collagen by Masson staining. Fourteen and 17 days after treatment, high-density, dense, and strongly stained collagen fibers were observed in the EPC treatment groups; the percentage of mature collagen was calculated by measuring the blue pixelated regions (×40). When the DB/+ EPC-treated group was compared with the control group, ^∗^*P* < 0.05 and ^∗∗^*P* < 0.01; when the DB/DB EPC-treated group was compared with the control group, ^#^*P* < 0.05 and ^##^*P* < 0.01.

To analyze the effect of EPC transplantation on wound healing, H&E staining and Masson staining were performed to evaluate epithelialization and deposition of collagen fibers in wound tissue, respectively. H&E staining results showed that the wounds treated with EPCs had extensive epithelial hyperplasia, thicker granulation tissue, and a large number of functional blood vessels containing red blood cells in the dermis, while the wound granulation tissue was thinner and had fewer functional blood vessels in the control group. The histological scores of DB/+ and DB/DB EPC-treated wounds were significantly higher than those of control wounds on day 7, while there was no difference between the two EPC-treated groups (Figure 6(a)). Masson staining showed that 7 days after injury, the amount of collagen fiber deposition in the wound tissues of the three groups was comparable (*P* > 0.05). Fourteen and 17 days after injury, the amount of collagen fiber deposition in the wound tissues of both the DB/+ EPC- and DB/DB EPC-treated groups was more abundant than that of the control group (*P* < 0.05). Fourteen days after injury, the amount of collagen fiber deposition in the wound tissue of mice in the DB/+ EPC-treated group was slightly more abundant than that in the DB/DB EPC-treated group, but the difference was not statistically significant (*P* > 0.05). Seventeen days after injury, the amount of collagen fiber deposition in the wound tissue of the DB/+ EPC-treated group was significantly more than that of the DB/DB EPC-treated group (*P* < 0.05) (Figure 6(b)).

### 3.5. Enhancement of Wound Angiogenesis by EPC Transplantation

**Figure 7:**
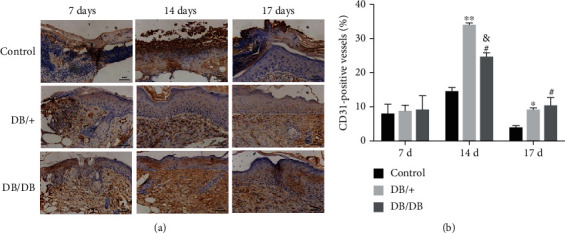
Immunohistochemical analysis of CD31 expression in wound tissues of the EPC-treated groups and the control group. The capillaries in the wound were visualized and quantitatively analyzed by CD31 antibody staining. Fourteen and 17 days after treatment, density and staining intensity of blood vessels were higher in the EPC-treated groups; wounds treated with EPCs had more CD31-positive blood vessels than those in the control group (×200). When the DB/+ EPC-treated group was compared with the control group, ^∗^*P* < 0.05 and ^∗∗^*P* < 0.01; when the DB/DB EPC-treated group was compared with the control group, ^#^*P* < 0.05 and ^##^*P* < 0.01; when the DB/DB EPC-treated group was compared with the DB/+ EPC-treated group, ^&^*P* < 0.05 and ^##^*P* < 0.01.

CD31 has been widely used as a marker to detect angiogenesis. To determine the effect of EPC transplantation on wound angiogenesis, the expression of CD31 was examined by immunohistochemistry. Our data showed that the difference in the CD31-positive rate in the wound tissue of the three groups was not statistically significant 7 days after injury (*P* > 0.05). However, 14 and 17 days after injury, the number of CD31-positive cells in the wound tissue of the two EPC-treated groups was higher than that of the control group (*P* < 0.05). Furthermore, the CD31-positive rate in the DB/+ EPC-treated group was higher than that of the DB/DB EPC-treated group 14 days after injury (*P* < 0.05), while 17 days after injury, the CD31-positive rate in both EPC-treated groups was comparable (*P* > 0.05) (Figure 7). These results suggest that local transplantation of EPCs can effectively promote the angiogenesis in the wound tissue.

### 3.6. mRNA Expression of Factors Significantly Related to the Biological Functions of EPCs

**Figure 8:**
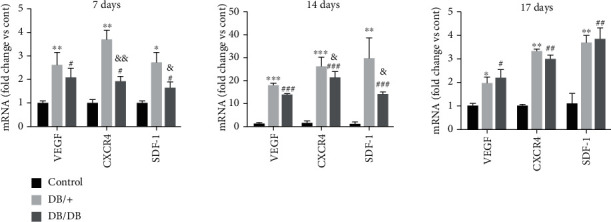
Expression levels of VEGF, SDF-1, and CXCR4 mRNA. As confirmed by real-time PCR analysis, the expression of VEGF, SDF-1, and CXCR4 mRNA in the wound tissues of the two kinds of EPC-treated groups was significantly increased compared with that in the control group on days 7, 14, and 17. When the DB/+ EPC-treated group was compared with the control group, ^∗^*P* < 0.05 and ^∗∗^*P* < 0.01; when the DB/DB EPC-treated group was compared with control group, ^#^*P* < 0.05 and ^##^*P* < 0.01; when the DB/DB EPC-treated group compared with DB/+ EPCs, ^&^*P* < 0.05 and ^&&^*P* < 0.01.

To further verify the potential effects of local transplantation of EPCs, we used Q-PCR to detect the mRNA expression of factors significantly related to the biological functions of EPCs. On day 7 after injury, the mRNA expression levels of VEGF, SDF-1, and CXCR4 in both the DB/+ and DB/DB EPC-treated groups were significantly higher than those in the control group (*P* < 0.05, *P* < 0.05, and *P* < 0.05). When the two EPC-treated groups were compared, the mRNA expression levels of CXCR4 and SDF-1 in the DB/+ EPC-treated group were higher than those in the DB/DB EPC-treated group (*P* < 0.01 and *P* < 0.05), and the mRNA expression levels of VEGF in the DB/+ EPC-treated group were slightly higher than those in the DB/DB EPC-treated group. However, the difference was not statistically significant (*P* > 0.05). On day 14 after injury, the mRNA expression levels of VEGF, SDF-1, and CXCR4 in the DB/+ EPC-treated group and DB/DB EPC-treated group were significantly higher than those in the control group (*P* < 0.005, *P* < 0.005, and *P* < 0.01), and the mRNA expression levels of CXCR4 and SDF-1 in the DB/+ EPC-treated group were higher than those in the DB/DB EPC-treated group (*P* < 0.05 and *P* < 0.05). On day 17 after injury, the mRNA expression levels of VEGF, SDF-1, and CXCR4 in the DB/+ EPC-treated group and DB/DB EPC-treated group were significantly higher than those in the control group (*P* < 0.05, *P* < 0.01, and *P* < 0.01), and the mRNA expression levels of VEGF, SDF-1, and CXCR4 in the DB/+ EPC-treated group and DB/DB EPC-treated group were similar (*P* > 0.05) (Figure 8).

### 3.7. Protein Expression of Factors Significantly Related to the Biological Functions of EPCs

**Figure 9:**
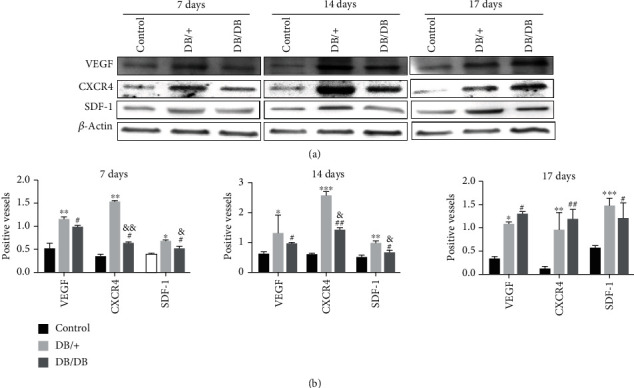
Expression levels of VEGF, SDF-1, and CXCR4 protein. As confirmed by western blot analysis, the protein expression of VEGF, SDF-1, and CXCR4 in the wound tissues of the two kinds of the EPC-treated groups was significantly increased compared with that in the control group on days 7, 14, and 17. When the DB/+ EPC-treated group was compared with control group, ^∗^*P* < 0.05 and ^∗∗^*P* < 0.01; when the DB/DB EPC-treated group was compared with the control group, ^#^*P* < 0.05 and ^##^*P* < 0.01; when the DB/DB EPC-treated group was compared with DB/+ EPCs, ^&^*P* < 0.05 and ^&&^*P* < 0.01.

Similarly, we used western blotting to examine the protein expression of factors significantly related to the biological function of EPCs. Our data showed that the protein expression levels of VEGF, SDF-1, and CXCR4 in both EPC-treated groups were significantly higher than those in the control group 7 days after injury (*P* < 0.05, *P* < 0.01, and *P* < 0.05). Consistent with the mRNA expression level, the protein expression levels of CXCR4 and SDF-1 in the DB/+ EPC-treated group were higher than those in the DB/DB EPC-treated group (*P* < 0.01 and *P* < 0.05), while the protein expression levels of VEGF were slightly higher than those in the DB/DB EPC-treated group, but the difference was not statistically significant (*P* > 0.05). Fourteen days after injury, the protein expression levels of VEGF, SDF-1, and CXCR4 in both the EPC-treated groups were significantly higher than those in the control group (*P* < 0.05, *P* < 0.01, and *P* < 0.05), and the expression levels of CXCR4 and SDF-1 in the DB/+ EPC-treated group were higher than those in the DB/DB EPC-treated group (*P* < 0.05 and *P* < 0.05). Likewise, the protein expression levels of VEGF, SDF-1, and CXCR4 in both the EPC-treated groups were significantly higher than those in the control group 17 days after injury (*P* < 0.05, *P* < 0.01, and *P* < 0.05), and the VEGF, SDF-1, and CXCR4 expression levels in the DB/+ EPC-treated group and DB/DB EPC-treated group were comparable (*P* > 0.05) (Figure 9).

## 4. Discussion

Currently, an increasing number of studies have demonstrated the effectiveness of cell therapy in skin wound repair/regeneration. Cell therapy involves the delivery of autologous or allogeneic cell components to the patient to repair or regenerate the damaged tissue caused by injury or disease, to correct the damage or lack of related cells, and ultimately to help improve or restore physiological functions [35]. Billingham and Reynolds first reported the study of skin cell transplantation for wound healing in 1952 [36]. Cell therapy for wounds can not only directly promote regeneration but also provide paracrine or nutritional signals to improve the molecular microenvironment of wounds and promote regeneration [1, 37]. This is why cell therapy is considered to be more effective than direct growth factor application. In addition, the limitations of direct treatment with cytokines and growth factors include the inherent low stability and short half-life of growth factors [38, 39] and the potential risk of single growth factor delivery. For example, the delivery of platelet-derived growth factor (PDGF) can increase the incidence of cancer mortality [40]. By using living cells, we can eliminate many problems associated with direct growth factor delivery and achieve better treatment outcomes.

Angiogenesis is one of the most important factors in the granulation formation and remodeling stage of wound healing [41] and can provide nutrition and oxygen to cells required for wound healing [42, 43]. EPCs are the key cell types involved in angiogenesis; they migrate to injury/ischemia sites and promote angiogenesis at the wound surface [44]. Although the vast majority of studies on EPCs have focused on the treatment of stroke [45] and other vascular diseases [46, 47], recent studies have suggested that EPCs may be used for skin wound healing.

Diabetic leg and foot ulcers are often associated with damages to the vascular system. Evidence exists that EPCs of diabetic patients are functionally defective [48, 49], which can manifest as impaired mobilization and homing, decreased proliferation potential, and increased apoptosis rate, and ultimately lead to a reduced number of EPCs and delayed wound healing [50]. Therefore, EPCs are a logical treatment choice for proangiogenic approaches. In recent years, EPCs have been shown to enhance skin wound healing by promoting the formation of new blood vessels in the body's granulation tissue [51]. Long-term culture of EPCs has been proven to maintain a high proliferation capacity, which can not only stably generate ECs with angiogenic potential in vitro [52] but also be directly integrated into new blood vessels [53]. Recent literature has shown that local application of amplified bone marrow-derived EPCs could increase the neovascularization of diabetic skin wounds by releasing cytokines in the local environment of the wound [51]. These results suggest that EPC transplantation may be beneficial in the treatment of skin wounds, especially chronic wounds that are often associated with reduced peripheral blood flow. Therefore, direct application of EPCs to these refractory wounds is an attractive treatment option.

Results of the previously published research showed that the best mobilization effect could be achieved by administering AMD3100+G-CSF to mobilize bone marrow EPCs [29]. In this study, we first mobilized EPCs into peripheral blood for in vitro isolation, culture, and amplification and then compared and analyzed the expression of functionally relevant molecules and biological behavior of EPCs derived from DB/DB and DB/+ mice. According to our results, DB/+ and DB/DB EPCs were both positive for Dil-Ac-LDL and FITC-UEA-1 staining. CD34, CD309, and CD133 in EPCs from the two sources were also positive based on flow cytometry, which is consistent with other reports [54]. This indicates that the cells isolated by gradient centrifugation of peripheral blood are indeed EPCs. In addition, the cell migration rate and tube diameter length of DB/+ EPCs were significantly higher than those of DB/DB EPCs, which was basically in accordance with the previous research of other scholars on the changes of EPC activity in diabetic state [55]. Furthermore, the mRNA and protein expression levels of VEGF, SDF-1, and CXCR4, which are strongly associated with the biological functions of EPCs, were significantly higher in DB/+ EPCs than in DB/DB EPCs. These results showed that the biological functions of DB/+ EPCs were more robust than those of DB/DB EPCs, which was consistent with the previous report that there were huge defects in the functional activity of EPCs in the diabetic state.

In addition, by locally transplanting EPCs into DB/DB mouse full-thickness skin defect wounds and comparing the therapeutic effects of DB/+ EPCs and DB/DB EPCs, we further explored the potential therapeutic effects of locally transplanted EPCs in promoting diabetic wound vascularization and healing. Collagen fiber deposition and granulation tissue are the key factors for wound healing, and CD31 has been widely used to detect angiogenesis. According to our results, compared with the NS control group, the CD31-positive rate, collagen deposition, and granulation formation in the wound healing process in the two EPC-treated groups were significantly improved, and the wound healing rate was significantly higher than that of the NS control group. However, there were no significant differences in the wound healing rate between the two EPC treatment groups. This is basically consistent with the results reported by other scholars that EPCs from healthy and diabetic donors have similar effects on wound healing in diabetic mice [56]. It can be speculated that diabetic patients can repair their wounds by transplanting autologous EPCs in vitro clinically, which provides new ideas for the treatment of clinical diabetic wounds. On the 14th day after transplantation, the angiogenesis ratio of the DB/+ EPC-treated group was significantly higher than that of the DB/DB EPC-treated group, but there was no difference on the 17th day. On the 17th day after transplantation, the collagen deposition ratio of the DB/+ EPC-treated group was significantly higher than that of the DB/DB EPC-treated group, but there was no difference on the 14th day. We speculate that EPCs derived from DB/+ mice may have short-term advantages in promoting vascular regeneration; thus, they exhibited stronger vascular regeneration ability at 14 days. However, as the wound was closed, blood vessels could no longer proliferate, and collagen was gradually deposited at this time. Although EPCs from DB/+ mice showed more abundant collagen synthesis on the 17th day, there was no significant difference as a whole in wound healing rate between the DB/+ EPC-treated group and the DB/DB EPC-treated group. One possible explanation for this observation is the injection site. Because the injection site of EPCs was in the basement membrane, it may have failed to achieve the desired effect on the growth of epidermal cells, which suggests that the depth of local injection of EPCs needs to be adjusted and explored. Furthermore, DB/+ EPCs may be short-acting and may be rejected as foreign bodies. The specific mechanism is worthy of further exploration.

SDF-1 is a chemokine in the CXCR family, and its chemotaxis is mediated by CXCR4. SDF-1 and CXCR4 can recruit relevant cells to promote wound healing through chemotaxis [57–59]. Studies have shown that SDF-1 is highly expressed in damaged organs and thus recruits circulating EPCs to reach damaged sites [60]. It has also been reported that the increased expression of CXCR4 and SDF-1 in wound tissue could promote the migration of vascular ECs; thus, it could promote wound healing in diabetic mice [61]. In addition, VEGF is crucial for angiogenesis. In this study, the expression levels of VEGF, SDF-1, and CXCR4 mRNA in wound tissues in both the DB/+ EPC-treated group and DB/DB EPC-treated group were significantly higher than those in the NS control group on days 7, 14, and 17 after injury. Among them, the CXCR4 and SDF-1 mRNA expression levels in wound tissues in the DB/+ EPC-treated group were significantly higher than those in the DB/DB EPC-treated group on days 7 and 14 after injury, while VEGF mRNA expression in the DB/+ EPC-treated group was slightly higher than that in the DB/DB EPC-treated group, but the difference was not statistically significant. Seventeen days after injury, the mRNA expression levels of VEGF, SDF-1, and CXCR4 in the DB/+ EPC-treated group and DB/DB EPC-treated group were similar. Furthermore, basically consistent with the mRNA expression, the VEGF, SDF-1, and CXCR4 protein expression levels in both the DB/+ EPC-treated group and DB/DB EPC-treated group were significantly higher than those in the NS control group on days 7, 14, and 17 after injury. CXCR4 and SDF-1 protein expression, but not VEGF, in the DB/+ EPC-treated group was significantly higher than that in the DB/DB EPC-treated group, and the protein expression levels of VEGF, SDF-1, and CXCR4 in the DB/+ EPC-treated group and DB/DB EPC-treated group were similar on day 17 after injury. Combined with the results of cell experiments, the expression levels of VEGF, SDF-1, and CXCR4 in diabetic EPCs were lower than those in normal EPCs. We speculate that EPCs derived from DB/+ mice may be short-acting; thus, there was no difference in the expression of VEGF compared with the DB/DB EPC-treated group, and the ability to promote the migration of vascular ECs to the wound surface was limited. Combined with previous reports in literature and existing experimental results, we speculate that local transplantation of EPCs may promote wound healing through the SDF-1/CXCR4 signaling axis. This is worthy of a further exploration.

In summary, whether it is obtained from a healthy donor or a diabetic donor, bone marrow-derived EPCs can be mobilized to the peripheral blood, isolated and cultured in vitro, and then locally transplanted to promote healing of diabetic wounds. We speculate that the functional activity of EPCs can be restored to a certain extent by in vitro culture, passaging, and removal from the high-sugar microenvironment of diabetes. Consequently, they can exert functional effects similar to EPCs derived from a healthy donor in terms of neovascularization, collagen deposition, granulation formation, and cytokine production; thus, they can improve the progress of chronic wound healing and increase the wound healing rate of diabetic mice at each time point. Therefore, local transplantation of EPCs into diabetic wounds may play a certain role in the wound repair process. In addition, EPC transplantation significantly promoted the expression of SDF-1, CXCR4, and VEGF. Local transplantation of EPCs may promote wound healing primarily through the SDF-1/CXCR4 signaling axis. Our results are valuable because they support the possibility of accelerating wound healing by isolating autologous EPCs from patients, which provides a new idea for the clinical treatment of diabetic wounds in the future. However, our current research has only observed superficial phenomena, and the specific molecular mechanism of action needs to be further studied in the future. In short, according to our experimental results, diabetic wounds may be repaired through autologous in vitro expanded EPC transplantation. The present findings will lay a practical foundation for the clinical application of autologous EPC cell therapy for chronic wound repair in the future.

## Figures and Tables

**Table 1:** Scoring of histologic parameters: score and features.

Score	Features
1±	Little epidermal and dermal organization, few capillary vessels, many infiltrated cells
2±	Moderate epidermal and dermal organization, newly formed capillary vessels in the entire wound area, few inflammatory cells
3±	Complete remodeling of the epidermis and dermis, well-formed capillary vessels, few inflammatory cells in perivascular or intravascular site

**Table 2:** qRT-PCR primer sequences used in this paper.

Gene	Forward	Reverse
CXCR4	GCTGACTGGTACTTTGGGAAAT	GAACGCTGCTGTAGAGGTTGA
SDF1	AGCATCTGAAAATCCTCAACACTCC	ACTTTAATTTCGGGTCAATGCACAC
VEGF	GAGCGGAGAAAGCATTTGTTT	CGAGTCTGTGTTTTTGCAGGA
GAPDH	CTTTGGCATTGTGGAAGGGCTC	GCAGGGATGATGTTCTGGGCAG

## Data Availability

The datasets used and/or analysed in the current study are available from the corresponding author upon reasonable request.

## Abbreviations

ECM: Extracellular matrix
KFs: Keloid fibroblasts
NFs: Normal fibroblasts.
TGF-*β*1: Transforming growth factor beta 1
EPCs: Endothelial progenitor cells
ECs: Endothelial cells
ECM: Extracellular matrix
AMI: Acute myocardial infarction
PDGF: Platelet-derived growth factor.
